# Performance evaluation of a PET insert for preclinical MRI in stand-alone PET and simultaneous PET–MRI modes

**DOI:** 10.1186/s40658-021-00415-1

**Published:** 2021-10-09

**Authors:** Gaelle Emvalomenos, Sofie Trajanovska, Binh T. T. Pham, Peter Doughty, Jerome Burnet, Isabelle Smith, Ruslan Garipov, Marie-Claude Gregoire, Nana Sunn, John McGrath, Steven R. Meikle

**Affiliations:** 1 Sydney School of Health Sciences, The University of Sydney, Camperdown, NSW 2050 Australia; 2 Brain and Mind Centre, The University of Sydney, 100 Mallett Street, Camperdown, NSW 2050 Australia; 3 Sydney Imaging Core Research Facility, The University of Sydney, Camperdown, NSW 2050 Australia; 4 MR Solutions, Guildford, UK; 5 School of Physics, The University of Sydney, Camperdown, NSW 2050 Australia; 6 Australian Nuclear Science and Technology Organisation, Lucas Heights, NSW 2234 Australia

**Keywords:** Preclinical PET, PET insert, PET/MRI, Simultaneous PET/MRI, NEMA

## Abstract

**Background:**

This study aimed to evaluate the performance of a preclinical PET insert in three configurations: as a stand-alone unit outside the MRI bore, inside the bore of a cryogen-free 3T MRI and, finally, while performing simultaneous PET/MRI studies.

**Methods:**

The PET insert consists of two rings of six detectors, each detector comprising 8 × 12 SiPMs reading out dual offset layers of pixelated LYSO crystals with a 1.4-mm pitch. The inner diameter is 60 mm, transaxial field of view (FoV) 40 mm and axial FoV 98 mm. Evaluation was based on NEMA NU 4-2008 guidelines with appropriate modifications. Spatial resolution and sensitivity were measured inside and outside the MR bore. Image quality, count rate and quantitative performance were measured in all three configurations. The effect of temperature stability on PET sensitivity during fast spin echo sequences was also evaluated. B_0_ field homogeneity and T1 and T2 relaxation times were measured using a water-filled phantom, with and without simultaneous PET operation. Finally, PET and MRI scans of a mouse injected with 10 MBq [^18^F]NaF and a mouse injected with 16 MBq [^18^F]FDG were performed in sequential and simultaneous modes.

**Results:**

Peak absolute sensitivity was 10.15% with an energy window of 250–750 keV. Absolute sensitivity values outside and inside the MR bore with MR idle agreed to within 0.1%. Outside the MR bore, spatial resolution was 1.21/1.59 mm FWHM (radial/tangential) 5 mm from the centre of the FoV which compared well with 1.19/1.26 mm FWHM inside the MR bore. There were no substantial differences between all three scan configurations in terms of peak NEC rate (175 kcps at 17 MBq), scatter or random fractions. Uniformity and recovery coefficients were also consistent between scanning modes. B_0_ field homogeneity and T1 and T2 relaxation times were unaltered by the presence of the PET insert. No significant differences were observed between sequential and simultaneous scans of the animals.

**Conclusions:**

We conclude that the performance of the PET insert and MRI system is not significantly affected by the scanning mode.

## Introduction

The combination of positron emission tomography (PET) and magnetic resonance imaging (MRI) has several advantages over other hybrid imaging approaches for preclinical [1] and clinical research. Firstly, MRI does not use ionising radiation and has better soft tissue contrast than CT. Secondly, MRI provides a wider variety of contrast mechanisms than other imaging modalities, enabling several different kinds of structural, functional or spectroscopic imaging in a single imaging session, all of which are highly complementary to the molecular specificity of PET [2, 3]. Finally, there are multiple ways in which the complementary information from PET and MRI can be combined for enhanced quantitative accuracy and reliability, including joint estimation of image and/or kinetic parameters [4]. However, this versatility also means there are many parameters to optimise, including when to acquire the data simultaneously versus sequentially in order to solve important biological and clinical problems.

There are three main hardware designs for PET/MRI systems [5]: (1) in-line PET and MRI scanners that enable sequential scanning, typically using a common patient pallet and carefully calibrated coordinate systems for accurate image registration; (2) fully integrated PET/MRI systems that exploit recent developments in MRI-compatible PET detector technology to enable simultaneous PET and MR scanning; (3) PET inserts for unmodified MRI systems, which use similar detector technology but can be removed from the MR bore to enable stand-alone PET imaging while freeing up time on the MRI for MR-only studies. To enable simultaneous PET/MRI, numerous challenges had to be overcome [5], including the effects of magnetic fields and radiofrequency (RF) signals on PET detectors [6–8]. On the other hand, PET detectors may cause inhomogeneity in the magnetic field that degrades the quality of the MR images [5, 9]. The sequential architecture minimises these challenges due to significant physical separation and shielding [10]. However, sequential PET/MRI has significant limitations, such as extended scan times, the potential for motion between studies and inability to acquire PET and MRI signals in parallel. Recently, MR-compatible silicon photomultipliers (SiPMs) have been developed which exhibit good quantum efficiency, high amplification gain and fast response times, making them well suited to simultaneous PET/MRI [11]. However, SiPMs can be somewhat sensitive to fluctuations in temperature necessitating careful temperature control in the MR environment [12]. SiPMs are now commonly used in new-generation PET inserts [11, 13–17] and fully integrated systems [18, 19]. For a thorough review of PET/MRI technology and challenges, the reader is referred to this review [7]*.*

The PET insert investigated in this study is a SiPM-based prototype, model I-402, customised for a 3T cryogen-free MRI (MR Solutions, Guilford, UK) dedicated to small-animal imaging. It is designed to operate inside the MR bore but can also be removed and used as a stand-alone unit on the benchtop. This provides a desirable level of flexibility in preclinical research studies. It also provides a unique opportunity to evaluate the effects of the MRI on performance of the PET insert (and vice versa) during simultaneous and sequential PET/MRI scanning compared with its performance in stand-alone mode. While the performance of various preclinical and clinical PET/MRI systems has typically been reported for two of these three acquisition modes [18, 20–23], there has not to our knowledge been a thorough evaluation in all three acquisition modes. Therefore, we evaluated the performance of the PET insert on the benchtop outside the MR bore, inside the MR bore, and inside the bore during simultaneous MR acquisition with a variety of pulse sequences. We followed the NEMA NU 4–2008 standard and adapted the tests when appropriate. We also evaluated the effect of PET detector electronics on MRI performance and investigated the effect of in-bore temperature fluctuations on PET sensitivity. Finally, we performed two PET/MRI animal studies to demonstrate performance of the system in simultaneous and sequential imaging modes with two common radiotracers.

## Materials and methods

### PET insert

**Fig. 1:**
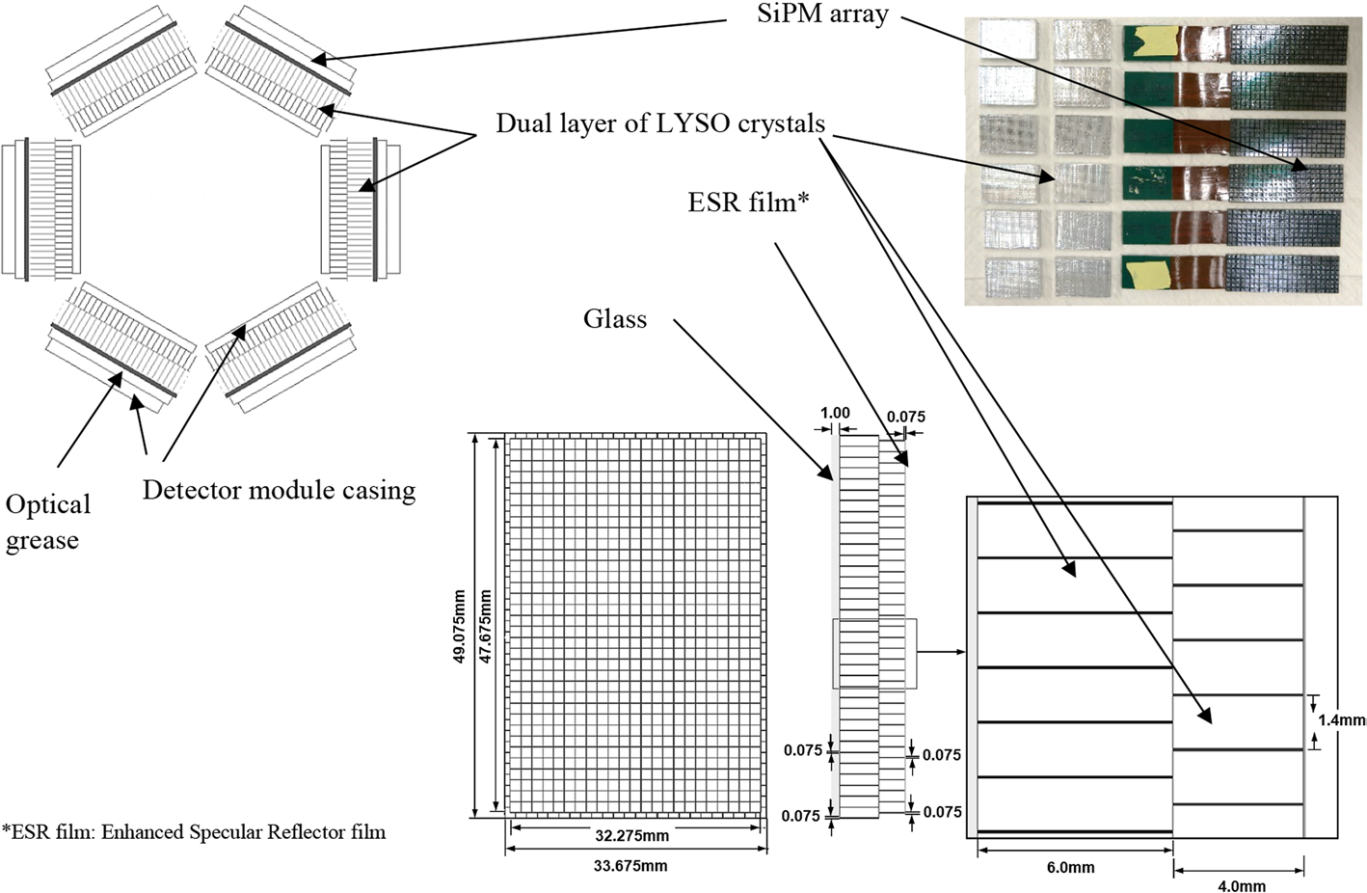
Schematic showing the arrangement of the detector modules and photograph of the SiPM and LYSO crystals

The PET insert, model I-402 (MR Solutions, Guildford, UK), is customised for a preclinical cryogen-free 3T MRI with a 17-cm-diameter bore and can also be used in the 7T MRI with a 24-cm-diameter bore (MR Solutions, Guildford, UK). All experiments in the current study were performed on the 3T MRI which is dedicated to mouse (body and brain) and rat (brain only) imaging. The PET insert has an inner diameter of 60 mm, a transaxial field of view (FoV) of 40 mm and an axial field of view (AFoV) of 98 mm. It consists of two rings of six detector modules, each detector comprising 8 × 12 SensL J-series TSV 3 mm SiPMs (SensL, Cork, Ireland) reading out a dual layer of pixelated 1.325 × 1.325 mm cerium-doped lutetium yttrium oxyorthosilicate (LYSO) crystals. The gaps between crystals are filled with 0.065 mm of ESR film for optical isolation and 10 μm of glue. The crystal array in the inner layer is a 34 × 23 matrix of 4-mm-thick LYSO, while the outer layer array is a 35 × 24 matrix of 6-mm-thick LYSO (Fig. 1, Table 1). The layers are offset by half the crystal pitch (0.7 mm) with respect to each other in order to determine the depth of interaction of the gamma photons and compensate for the parallax effect [24, 25]. The bottom crystal layer is coupled with the SiPM sensor via a 1-mm glass diffuser and a thin layer of BC-630 optical grease (Saint Gobain, https://www.crystals.saint-gobain.com/sites/imdf.crystals.com/files/documents/detector-assembly-materials_69673.pdf). This architecture forms the detector module.

**Table 1:** Specifications of the PET insert

Characteristics	Value
**System**	
Number of detectors per ring	6
Number of rings	2
Total number of crystals	18,336
Transaxial FoV (mm)	40
Axial FoV (mm)	98
Coincidence time window (ns)	12
Energy window (keV)	250–750
**Detector module**	
Scintillators	LYSO
Crystal array: bottom layer	35 × 24 (6 mm thick)
Crystal array: top layer	34 × 23 (4 mm thick)
Crystal pitch (mm)	1.4
Scintillator dimension (mm)	1.325 mm pixel
Total crystal dimensions (mmxmm)	49 × 33.6 (10 mm thick)
SiPM array	12 × 8
SiPM size (mmxmm)	50.2 × 33.6
SiPM pitch (mm)	4.2 (3-mm sensor pads and 1.2-mm gap between them)

Event positioning is performed using a centre of gravity (CoG) method [26]. Each detector is connected via a long rigid flexi board to its event capture board (located outside the magnet), which amplifies, shapes and digitises the signals. A coarsely sampled fast signal from the SiPM array is used for triggering and timing information, and each single event is time stamped. A slower, more finely sampled signal is used to determine the position and energy of the event. The digitised data are passed to the central Controller Board which processes the coincidences using the timestamp information from the singles, with ADC sampling resolution of 0.8 ns per bin, coincidence timing resolution of 4.8 ns and a coincidence window of 12 ns. The final coincidence pairs are then transferred from the Controller Board to the host capture PC via a 1GBit ethernet cable.

**Fig. 2:**
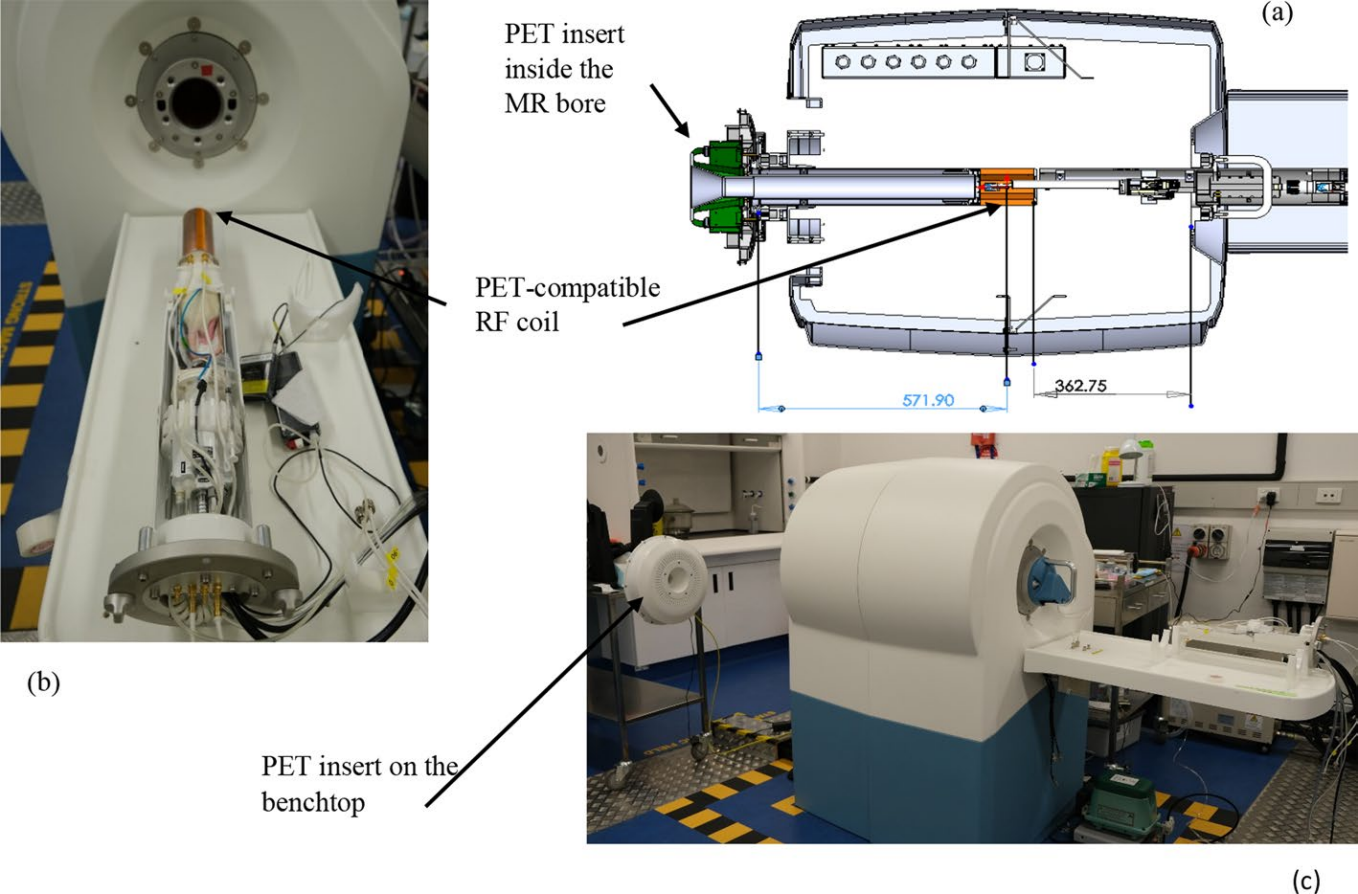
**a** Schematic of the 3T MRI bore and PET insert and **b** photographs of the MR-compatible animal bed, **c** 3T MRI and PET insert outside the bore

The system is very compact, with most electronics positioned outside the MR bore (Fig. 2). Copper foil was placed along the whole length of a plastic tube for radio frequency shielding from the MRI. This tube connects the detectors to the external electronics where the power supply and ethernet cable are located. A 250–750 keV energy window was used for this study. Data are acquired in a list-mode format. Prompt and random events are saved, and the delayed window method is used to estimate randoms. Scatter events are estimated using scatter fraction tables based on energies [27, 28]. True coincidences are calculated by subtracting randoms and scatters from prompt sinograms, and then attenuation corrected using MR-based attenuation correction prior to image reconstruction. The attenuation map is created using a noise-filtered/thresholded T1-weighted MR image. A binary map is used, where only air and water are defined, without more detailed segmentation of fat and bone, which is sufficient for small-animal applications. Two reconstruction algorithms are available: 2D-filtered back-projection (FBP) which is useful for real-time reconstruction and NEMA tests and 3D-ordered subset expectation maximisation (OSEM) [29].

### Performance evaluation

#### Spatial resolution

A 0.5 MBq ^22^Na point source was positioned in the centre of the transaxial FoV, with radial offsets of 0, 5, 10 and 15 mm. The 25 mm source offset specified by NEMA is beyond the 40 mm transaxial FoV of this PET insert and, therefore, could not be measured. These measurements were taken at the centre and at one-fourth of the AFoV and were repeated in the MR bore and outside the MR bore. Data were collected for 3 min in each position (> 10^5^ prompt counts per measurement). The images were reconstructed with 2D FBP (voxel size 0.35 × 0.35 × 0.35 mm^3^, a quarter of the crystal pitch) from 2D sinograms generated using single-slice rebinning (SSRB) with a slice thickness of 4.2 mm.

We also imaged a micro-resolution phantom (inner height 1.5 cm, inner diameter 2.2 cm, rod diameters 0.7, 0.8, 0.9, 1.0, 1.1 and 1.2 mm, centre-to-centre spacing twice the rod diameter) filled with 10 MBq ^18^F. Data were acquired for 20 min in all three configurations and in two positions: at the centre of the AFoV and at ¼ of the AFoV. We reconstructed the data with 32 subsets and 5 iterations of 3D-OSEM and voxel size of 0.35 × 0.35 × 0.35 mm^3^ (a quarter of the crystal pitch) and no smoothing. These parameters are similar to those used for animal imaging with the exception of attenuation correction, as the MR-based attenuation correction is unable to account for perspex which lacks a proton signal.

#### Sensitivity

The same ^22^Na point source was positioned in the centre of the transaxial FoV and stepped along the axis of the PET insert with an increment of half the crystal pitch, 0.7 mm, with the PET insert outside the MR bore. Two-minute scans were acquired at each position, and background true event rates were determined with no source in the PET insert for the same duration. 2D sinograms were generated using the SSRB method according to NEMA standards. The count rate was determined by dividing the masked sinogram summed over the acquisition time. The same method was applied to the background scan. The absolute sensitivity at each position was calculated as:

1 $${\text{Absolute sensitivity}} = \frac{{\left( {R - B} \right) \times 100}}{\gamma A}$$

where *R* is the point source count rate, *B* is the background rate, *A* is the activity and *γ* is the branching ratio of ^22^Na.

Since subjects or phantoms need to be loaded manually into the system, i.e. there is no automatic positioning, it was not feasible to repeat the entire axial sensitivity measurement with the PET insert inside the MR bore. This would have required manual repositioning of the point source at every axial location with a precise increment of half the crystal pitch. Instead, one absolute sensitivity measurement was taken inside the MR bore, approximately at the centre, and compared with a measurement taken outside the MR bore in the same position enabling accurate comparison of the sensitivities under these two conditions.

#### Count rate performance and scatter fraction

The noise equivalent count rate (NECR) was measured using a mouse-sized NECR phantom (70 mm long, 25 mm diameter) in all three configurations. The line source of the phantom was filled with ≥ 30 MBq of ^18^F for the three studies, and the duration of scans was approximately 22 h to allow activity to decay to approximately 0.1 MBq. For the simultaneous study, a series of echo planar imaging (EPI) sequences was acquired throughout the 22-h PET scan. In all three configurations, the phantom was placed in the PET-compatible mouse body coil. For all scans, prompt, random and scattered 2D sinograms were generated using SSRB [30]. Rates of true (*T*), random (*R*) and scattered (*S*) coincidences were determined, and NECR was calculated as:

2 $${\text{NECR}} = \frac{{T^{2} }}{T + S + 2R}$$

#### Image quality

The NEMA image quality (IQ) phantom was used to assess quantitative accuracy and overall image quality. The phantom has five fillable rods of diameter 1, 2, 3, 4 and 5 mm in a cold background, a uniform warm background area and two cold cylinders in a warm background, one filled with water and one with air. Three scans were performed in the following order: PET insert outside the MR bore, PET insert inside the MR bore and simultaneous PET/MRI. The first scan was 20 min with 3.7 MBq of ^18^F at the start of acquisition; then, without refilling the phantom, we adjusted the duration to 22 min and 28 min for the 2nd and 3rd scans, respectively, to compensate for radioactive decay. For both scans inside the MR bore, the phantom was placed inside the mouse body RF coil, whereas for stand-alone the coil was removed. MR sequences used during the simultaneous scan were T1-weighted coronal and axial scans. Images were reconstructed using 5 iterations and 32 subsets of 3D OSEM with voxel size 0.35mmx0.35mmx0.35 mm. The image reconstruction was run with all corrections applied, excluding attenuation correction as the MR-based attenuation correction is unable to create an attenuation map for the perspex component of the phantom which lacks a proton signal.

#### Quantitative accuracy with dead time correction

We used a 5-mL syringe phantom to evaluate the accuracy of dead time correction and performed three scans: PET outside the bore, PET in the MR bore and simultaneous PET/MR. At least 35 MBq of ^11^C activity was present in the syringe at the start of each two-hour scan. For the simultaneous PET/MRI scan, we repeated fast spin echo (FSE) T1-weighted sequences for coronal and axial imaging. All scans were reconstructed using 3D-OSEM with 1 iteration and 16 subsets and voxel size 0.7 mm × 0.7mmm × 0.7 mm. All corrections were applied. Measured PET coincidence rates were determined from ROIs covering the whole syringe. At low activity (up to 2 MBq), dead time was considered negligible. A straight line was fitted to these data points, extrapolated to higher count rates and used to calculate the expected true coincidence rates. A non-paralyzable dead time model was fitted to the PET data:

3 $$R_{O} = \frac{{R_{t} }}{{1 + R_{t} \tau }}$$

where *R*_*O*_ is the observed coincidence rate, *R*_*t*_ is the true coincidence rate and *τ* is the dead time. Nonnegative linear least squares was used (MATLAB, MathWorks, USA) to estimate *τ*.

#### Temperature stability

To evaluate temperature stability of the SiPM detectors, we adjusted the temperature of the gradient chiller in small increments (1 degree initially and later 0.5 degree steps) from 18.5 to 32 °C. We waited for the gradient/temperature sensors to reach their new stable set limit (approximately 7–8 min after temperature adjustment) and collected 3 min PET data at each temperature reading. At each new setting we read the SiPM head temperature (average and standard deviation) from the log files.

#### Impact of PET insert on MRI performance

The signal-to-noise ratio, B_0_ inhomogeneity, T1 and T2 relaxation times of the MRI were evaluated with PET acquiring data and with the PET insert idle, using a water phantom positioned in the PET-compatible mouse body coil.

#### Animal studies

Animal studies were conducted with the approval of the University of Sydney Animal Ethics Committee (AEC). Two female mice (approximately 30 g) were anaesthetised with isoflurane (1.5–3%), and their respiration rate (RR) and body temperatures were continuously monitored. The RR was 40–50/min throughout the scan. Body temperature was maintained between 35 and 37 °C. One mouse was injected with 16.3 MBq of [^18^F]FDG into the tail vein 30 min before starting the scan. A 30-min simultaneous PET/MRI scan (T1-weighted and T2-weighted coronal images) was acquired, followed by a 10-min PET scan and, finally, T1-weighted and T2-weighted coronal MRI scans. The other mouse was injected with 9.8 MBq of [^18^F]NaF via the tail vein 30 min before starting the scan. A 30-min simultaneous PET/MRI scan (T1-weighted and T2-weighted coronal images) was acquired, followed by a 15-min PET scan and finally T1-weighted and T2-weighted coronal MRI. The images were reconstructed using 32 subsets and 5 iterations of 3D-OSEM with a voxel size of 0.35 × 0.35x0.35 mm after all corrections were applied. AMIDE software (http://amide.sourceforge.net/) was used to align manually the PET image on the corresponding MR image.

## Results

### Spatial resolution

Table 2 shows the measured tangential, radial and axial components of spatial resolution at the centre and at ¼ of the AFoV with the PET insert positioned inside the MR bore and outside the bore. At ¼ AFoV, all three components of spatial resolution were approximately 1.2 mm FWHM at 5 mm radial offset and were not significantly different inside and outside the MR bore. These values translate into volumetric resolutions (tangential × radial × axial) of 1.7 mm^3^ inside the MR bore and 2.5 mm^3^ outside the bore. There was only minor degradation in spatial resolution at 15 mm radial offset where all three components were approximately 1.4 mm FWHM and volumetric resolution was approximately 2.8 mm^3^, with only minor differences inside and outside the MR bore. Results at the axial centre of the FoV were similar except that the axial resolution was noticeably degraded to > 2 mm. We attribute this to the fact that the point source was positioned exactly between the two detector rings and, together with the hexagonal geometry of the PET insert, leads to poor sampling at the centre of the FoV. However, no significant differences were observed between spatial resolution measured with the PET insert positioned inside the MR bore and outside the bore.

**Table 2:** Spatial resolution at 1/4 axial offset from the centre of the AFoV and spatial resolution at the centre of the FoV

Offset (mm)	Tangential (inside the MRI)	Tangential (outside the MRI)	Radial (i)	Radial (o)	Axial (i)	Axial (o)
*FWHM (mm) at 1/4 AFoV						
0	1.06	1.43	1.1	1.32	1.14	1.03
5	1.21	1.58	1.15	1.31	1.25	1.22
10	1.29	1.26	1.46	1.3	1.55	1.11
15	1.36	1.43	1.46	1.43	1.4	1.2
**FWTM (mm) at 1/4 AFoV						
0	2.36	2.77	3.28	3.11	2.55	2.39
5	2.05	2.55	4.43	3.05	2.48	2.62
10	2.78	2.85	3.43	4.37	2.73	2.77
15	2.88	5.19	3.93	4.93	3.09	2.85
**FWHM (mm) at the centre of AFoV**						
0	1.46	1.09	1.71	1.54	1.61	1.99
5	1.26	1.59	1.19	1.21	2.52	2.39
10	1.61	1.28	1.53	1.51	2.28	1.99
15	1.32	1.65	1.29	2.04	1.47	2.28
**FWTM (mm) at the centre of AFoV**						
0	2.81	2.02	4.65	3.51	3.69	3.49
5	2.64	3.39	2.22	4.02	3.51	3.99
10	3.7	2.56	2.59	3.75	3.95	2.96
15	3.03	2.84	3.17	4.16	3.73	3.56

^*^FWHM: full width at half maximum

**FWTM: full width at one tenth of the maximum

**Fig. 3:**
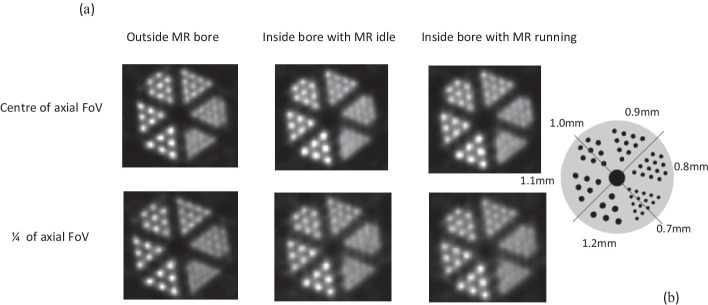
**a** Reconstructed images of the micro-resolution phantom obtained with 3D OSEM (32 subsets, 5 iterations) in different configurations (AMIDE software). **b** Schematic of the micro-resolution phantom with rod diameters ranging from 0.7 to 1.2 mm

Reconstructed images of the micro-resolution phantom are shown in Fig. 3a. There is no noticeable difference between images in the three configurations at the centre of the AFoV or at ¼ of the AFoV. In all three cases, the 0.9-mm rods were resolved after the second iteration of 3D OSEM (32 subsets), whereas five iterations were needed to resolve the 0.8-mm rods.

### Sensitivity

**Fig. 4:**
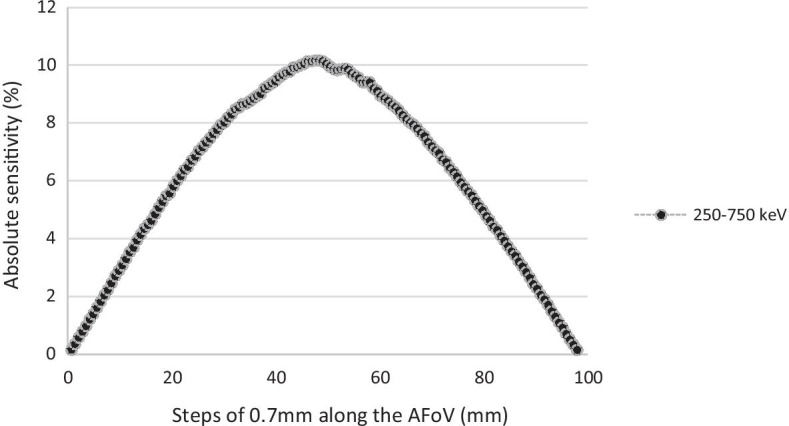
Axial sensitivity profile for an energy window of 250–750 keV, PET insert outside the MR bore

The sensitivity profile for the PET insert outside the MR bore followed a characteristic triangular shape (Fig. 4). Peak system absolute sensitivity was 10.15% for the energy window 250–750 keV. Absolute sensitivity was 9.55% inside the MR bore and 9.41% outside the MR bore with the point source located at the same position close to the centre of the AFoV.

### Count rate performance and scatter fraction

**Fig. 5:**
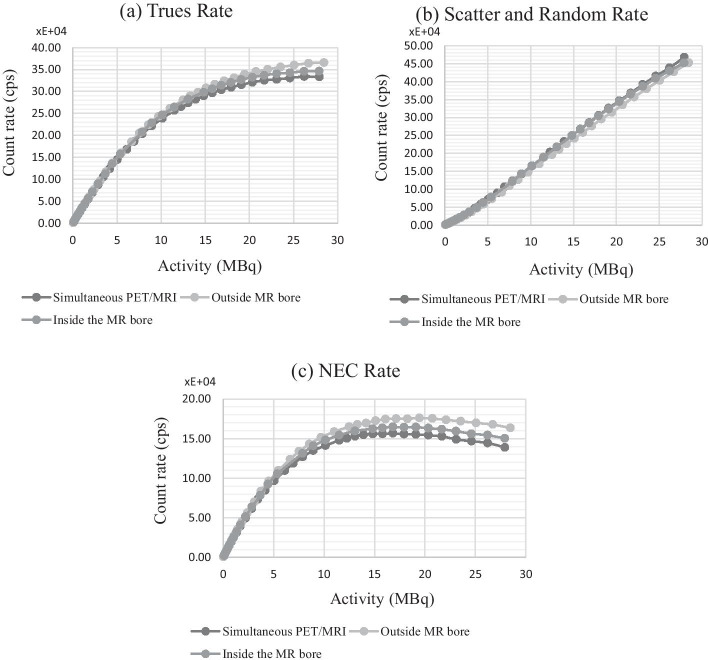
The **a** trues rate, **b** scatter and randoms rate, and **c** NEC rate in counts per second plotted against activity in MBqs for scans done with PET outside the MR bore, PET inside the MR bore and simultaneous PET/MR. The mouse size NEMA NEC rate phantom was used

Figure 5 shows the trues, scatter and randoms rates along with NECR as a function of source activity. Total coincidence rates were consistent across the three scanner configurations, and the trues rates were similar in the linear response region which corresponds to the activities used routinely in animal imaging. However, for activity ≥ 15 MBq, when compared to the acquisition outside the bore, the trues rate was approximately 4.5% lower inside the bore when the MR was idle and a further 8% lower during simultaneous imaging (Fig. 5a). Scatter and randoms rates for the two scans acquired inside the MR bore were approximately 3% higher than those measured outside the bore (Fig. 5b). The NECR peak occurred at approximately 17 MBq for all three scan configurations (Fig. 5c). However, peak NECR was approximately 6% lower inside the bore with the MR idle and 10% lower with the MR active compared with outside the bore.

### Image quality

**Fig. 6:**
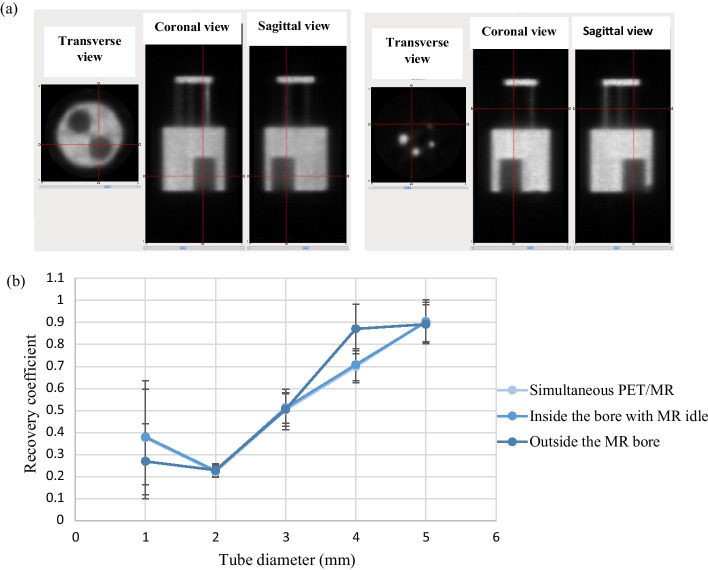
**a** PET images of the image quality (IQ) phantom (AMIDE software), **b** recovery coefficients for tubes with diameters ranging from 1 to 5 mm. Error bars represent standard deviation

Figure 6a shows PET images of the NEMA IQ phantom. Uniformity, spillover ratios (Table 3) and recovery coefficients (Fig. 6b) were not significantly different between the three scan configurations.

**Table 3:** (a) Spillover ratios (SOR) for the water- and air-filled regions of the IQ phantom, (b) uniformity measurements for the IQ phantom for the three scanner configurations, presented in NEMA NU 4-2008 format

(a) Region	Scanner configuration	SOR	%STD
Water-filled cylinder	Simultaneous PET/MR	0.28	8.2
	PET inside the MR bore	0.28	7.7
	PET outside the MR bore	0.24	9.2
Air-filled cylinder	Simultaneous PET/MR	0.35	7.2
	PET inside the MR bore	0.36	7.1
	PET outside the MR bore	0.35	8.5

(b) Scanner configuration	Mean (Uniformity)	Maximum (Uniformity)	Minimum (Uniformity)	%STD
Simultaneous PET/MR	0.0071	0.0084	0.0052	4.2
PET inside the MR bore	0.0070	0.0081	0.0054	4.1
PET outside the MR bore	0.0042	0.0049	0.0033	4.3

### Quantitative accuracy with dead time correction

Dead time was estimated to be 100 ns when positioned outside the MR bore, 122 ns when inside the bore with the MR idle and 140 ns for the simultaneous PET/MRI scan. Dead time correction using a non-paralyzable dead time model with τ = 122 ns resulted in < 6% bias in corrected count rates when activity in the FoV was ≤ 30 MBq, which covers the typical activity range for rat brain and mouse whole body scans.

### Temperature stability

A strong linear correlation (*r* = 0.99) between sensitivity and temperature was observed. The change in sensitivity was only 3% within the typical temperature range observed during PET/MRI scans (15–20 °C) when using fast MR sequences.

### Impact of PET insert on MRI performance

B_0_ inhomogeneity was − 181.77 ± 9.83 Hz with the PET insert acquiring data in the background and − 191.07 ± 10.73 Hz with the PET insert idle. The relaxation times for T1-weighted acquisition were 2.66 ± 0.64 s with PET running and 2.65 ± 0.79 s with PET idle, while for T2-weighted acquisition they were 811.27 ± 63.75 ms and 832.54 ± 90.12 ms, respectively.

### Animal studies

**Fig. 7:**
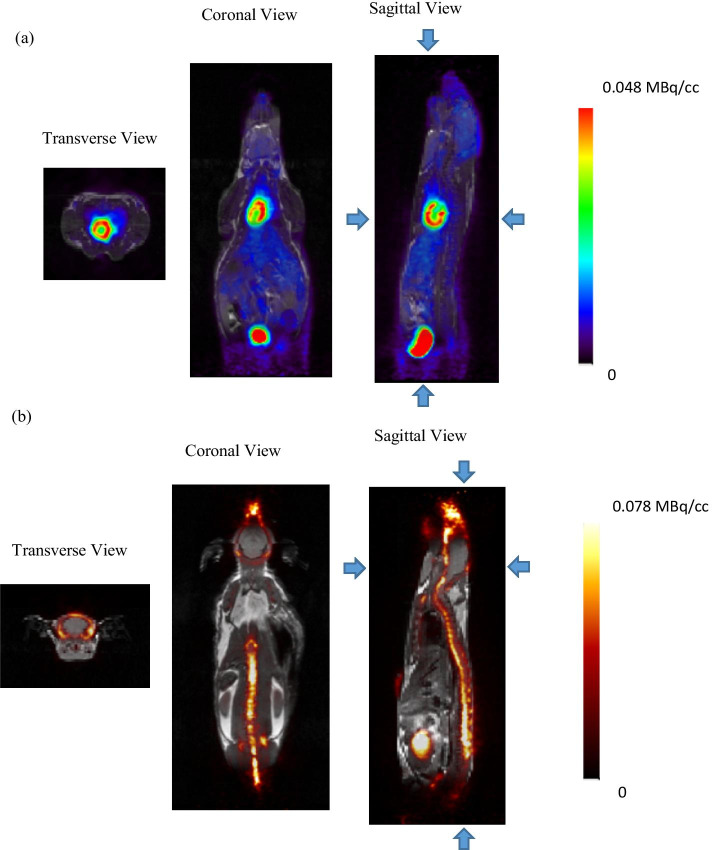
**a** Whole body mouse [^18^F]FDG PET/MRI images, 30 min PET acquired simultaneously with T1-weighted coronal MRI. **b** Whole body mouse [^18^F]NaF PET/MR images, 30-min PET acquired simultaneously with T1-weighted coronal MRI (AMIDE software)

Figure 7a, b shows the co-registered PET/MRI images acquired simultaneously for the [^18^F]FDG and [^18^F]NaF scans, respectively, demonstrating excellent image quality for both modalities. Typical biodistribution is observed with uptake in the myocardium, liver and brain in the case of [^18^F]FDG, uptake in the spinal column, tail vertebrae and ribs in the case of [^18^F]NaF, and excretion via the kidneys in both cases.

## Discussion

The small-animal PET insert evaluated in this study was designed to allow simultaneous imaging with preclinical 3T and 7T MRI systems, while also retaining the flexibility to be used as a stand-alone unit on the benchtop. While previous studies have investigated the potential impact of interference between the two modalities during simultaneous acquisition [18, 20–23], the present study provides a thorough performance evaluation in all three configurations: PET insert outside the MR bore, inside the MR bore with the MR idle and during simultaneous acquisition of MR sequences.

There was no significant difference between the sensitivity of the PET insert inside and outside the MR bore. The relatively high sensitivity of 10.15% achieved by the system can be attributed to its geometry, i.e. the long AFoV and narrow ring diameter, as well as the high detection efficiency of the dual-layer LYSO detectors. This sensitivity compares favourably with other MR-compatible PET inserts, such as the Bruker Si 103 (Bruker Corporation, Billerica, USA) with 11% absolute peak sensitivity, and dedicated small-animal PET systems, such as the nanoScan PET/CT (Mediso Medical Imaging Systems, Hungary) with 8.41% absolute peak sensitivity [10], which have similarly long axial fields of view and employ similar detector materials. This value is comparable as well with the absolute peak sensitivity of the MR Solutions PET insert (model 80-2), 7.9% (measured inside the MR bore without RF coil), that has a longer axial FoV, 103 mm, but a wider inner diameter bore, 116 mm [23] (Table 4).

**Table 4:** Comparison with other PET/MRI systems

System	MADPET4 [14]	MR Solutions PET insert model I-802 [23]	MR Solutions PET insert model I-402 (present study)
**MRI** (Tesla)	7	7	3
**PET characteristics**			
Energy window (keV) ^ #^energy threshold, time-over-threshold method	250^#^	250–750	250–750
Internal ring diameter (mm)	88	116	60
Axial FoV (mm)	20	103	98
Maximum sensitivity (%)	0.7	7.9*/7.5**	10.15^˫^
Tangential spatial resolution Centre FoV (mm)	1.29	1.69**	1.09^˫^
Radial spatial resolution Centre FoV (mm)	1.29	1.74**	1.54^˫^
Peak Mouse NECR (MBq)	29	34*/35**	17^˫^
Peak Mouse NECR (kcps)	103	427*/300**	175^˫^

^*^Without RF coil inside the MR bore

^**^With RF coil inside the MR bore

^˫^Outside the MR bore

The possible effect of the static magnetic field on spatial resolution was tested by taking measurements outside the MR bore and inside the MR bore with the MR idle. Spatial resolution was not significantly affected by the presence of the magnetic field. This is probably because of the relatively low static magnetic field of our 3T system. At higher field strengths, the positron range is slightly reduced in directions orthogonal to B_0_ (transverse and radial) leading to improved in-plane spatial resolution [31, 32]. However, axial elongation of positron range is also observed which degrades axial resolution and introduces artefacts [33]. Improvements in spatial resolution are also dependent on the energy of the positron emitter [34]. In future, we will repeat this study in the 7T system to see whether we observe these effects at higher field strength. The measured radial/tangential and axial components of spatial resolution were < 1.2 mm near the centre of the FoV which compares favourably with the MADPET4 [14] and MR Solutions PET insert 80-2 [23] (Table 4) but also with other current-generation small-animal PET/MRI systems [11, 16, 35]. Additionally, radial resolution was relatively uniform over 5–15 mm radial offset from the centre of the FoV, indicating that the DOI method implemented on this system is effective at minimising resolution degradation due to parallax error. Beyond the NEMA guidelines, a micro-resolution phantom was imaged in the three different configurations at the centre of the AFoV or at ¼ of the AFoV to further test the spatial resolution performance. In all three cases, by using 3D OSEM reconstruction the 0.9-mm rods were resolved after two iterations and 0.8-mm rods were resolved after five iterations, slightly below the performance of similar systems that resolved the 0.7-mm rod with iterative reconstruction methods [16, 35].

Studies performed with the IQ phantom demonstrated that there is no significant difference in quantitative accuracy between scans performed outside or inside the MR bore or during simultaneous imaging. There was no significant difference between the uniformity of the phantom inside the bore and outside the bore, with the observed differences being within experimental error. The consistency in uniformity and spillover ratios suggest that scatter correction accuracy is not affected by the magnetic field or pulsing of RF gradients. Similarly, the consistency of recovery coefficients across scanner configurations suggests that there is no impact of the static magnetic field or RF pulsing on partial volume effects, consistent with our spatial resolution results. Thus, the same scatter correction can be applied in any configuration for low-energy radioisotopes such as ^18^F. A bubble was present inside the 1-mm rod of the IQ phantom, which prevented accurate calculation of the recovery coefficient for this rod. However, since the IQ phantom was not refilled between scans, results can be compared across the three configurations studied. The difference in RC of the fourth rod is within experimental error.

Concerning the scatter and random rates, the increase observed inside the bore can be explained either by higher backscatter from the magnet or by impacts of the gradient fields and RF pulsing on the timing resolution of the detectors. Degraded timing resolution would increase the number of random events registered as coincidences and, conversely, decrease the number of true coincidences registered within the coincidence time window. The combination of increased scatter and randoms rates and decreased trues rate explains the decrease in NECR seen inside the MR bore, and during simultaneous PET/MR (at high count rates). The NECR of the MR Solutions PET insert 80-2 presented a 30% decrease when measured inside the MR bore with the RF coil in comparison with the NECR measured without the RF coil [23], (Table 4). The NECR of the GE SIGNA clinical PET/MR [18] when the MR was running was also found to be lower than the NECR when the MR was idle. Grant et al*.* [18] and Deller et al*.* [20] identified interference of the RF pulsing on the PET electronics as the cause of longer dead time, and therefore, loss of counts: this would explain the decreased NECR when the MR is active. This could also explain our results but does not explain the difference observed between the NECR outside the bore and inside the bore with no RF pulsing. Increased backscatter may explain the difference in these configurations. In addition, the SiPMs may behave slightly differently since there is less shielding from light outside the bore, and SiPMs are very sensitive to light. In general, the peak NECR of 175 kcps at 17 MBq compares favourably with other SiPM-based systems [11, 16, 23, 35].

At typical activity concentrations, the differences in count loss due to dead time in the three configurations were negligible and dead time correction using a non-paralyzable model and a single dead time parameter of 122 ns was sufficiently accurate. Previous studies of simultaneous PET/MR systems have shown that RF leakage into PET electronics results in an increase in dead time [18, 20]. Imperfections in the shielding of PET electronics may account for the differences we observed at higher count rates. The strength and duration of the RF pulses depend on the MR imaging sequence chosen. Therefore, a further investigation with different MR sequences and their effect on dead time may be warranted. However, this effect does not explain the small difference in dead time we observed between scans done outside and inside the MR bore with the MR idle. A possible explanation might be increased backscatter from the magnet causing an increase in the scatter count rate. It should be noted that these small dead time differences can be incorporated into the dead time correction for more accurate quantification at very high activities.

The PET insert exhibited good temperature stability in the range of 15–30 °C. Even during fast MRI sequences (EPI or TOF 3D), which typically raise the temperature of the gradients, and with the gradient chiller temperature at 18.5 °C, the SiPM temperatures never reached > 20 °C. This is an important result as SiPMs are known to be affected by environmental temperature [36].

We did not perform animal studies on the benchtop due to the lack of portable physiological monitoring systems during these experiments. PET images acquired with the MR idle and during simultaneous imaging were highly comparable for both radiotracers. Taken all together, the NEMA and non-NEMA experiments demonstrate that the performance of the PET insert is not affected by being operated inside the MR bore or as a stand-alone unit on the benchtop. This flexibility offers some practical advantages. First, it allows more efficient use of the PET insert in a busy preclinical imaging laboratory since the PET insert can be used on the benchtop concurrently with MR-only studies. Second, more complex PET studies requiring, for example, microdialysis or voltammetry, may need to be performed outside the MR bore because of either space or MR compatibility issues with ancillary instrumentation. Finally, in other situations it may be desirable to perform PET scans inside the MR bore, enabling use of complementary information from the two modalities to improve the accuracy and reliability of parameter estimates [4].

Table 4 compares the key NEMA standards measurements between similar PET/MRI systems, namely the PET insert presented in the current study, model I-402, the MR Solutions PET insert, model I-802 [23] and the MADPET4 [14].

## Conclusions

The performance of an MR-compatible PET insert coupled to a 3T preclinical MRI, in terms of spatial resolution, sensitivity and count rate, was not significantly different when used as a stand-alone unit outside the MR bore and when used inside the MR bore with or without the MR pulsing. Similarly, B_0_ field homogeneity and T1 and T2 relaxation times were unaffected by the presence of the PET insert. The PET insert exhibited good temperature stability and accuracy of dead time correction in different configurations, and we demonstrated the capability of the system in typical animal studies. The stability of its performance enables the PET insert to be used in several different contexts according to the requirements of the study. In addition to enabling simultaneous PET–MR imaging, the PET insert can be used as a stand-alone unit on the benchtop which allows the use of non-MR-compatible instrumentation and provides for efficient workflows.

## Data Availability

The data sets generated during and/or analysed during the current study are available from the corresponding author on reasonable request.
